# Taxonomic and Functional Dynamics of Bacterial Communities During Drift Seaweed Vermicomposting

**DOI:** 10.3390/microorganisms13010030

**Published:** 2024-12-27

**Authors:** Manuel Aira, Ana Gómez-Roel, Jorge Domínguez

**Affiliations:** Grupo de Ecoloxía Animal (GEA), Universidade de Vigo, E-36310 Vigo, Spain; ana.gomez.roel@uvigo.gal (A.G.-R.); jdguez@uvigo.gal (J.D.)

**Keywords:** earthworms, seaweed, bacterial communities, 16S RNA, bacterial functionality, vermicompost

## Abstract

Seaweed is a valuable natural resource, but drift or beach-cast seaweed is considered a waste product. Although seaweed is traditionally used as an organic amendment, vermicomposting has the potential to transform the material into valuable organic fertilizer, thereby enhancing its microbial properties. This study aimed to investigate the dynamics of the taxonomic and functional bacterial communities in seaweed during the vermicomposting process by high-throughput sequencing of 16S rRNA gene amplicons. Vermicomposting changed the composition of the bacterial communities, as indicated by the low proportion of bacterial taxa common to the bacterial communities in the raw seaweed and vermicompost (21 to 56 ASVs from more than 900 ASVs per sample type). The observed increase in taxonomic diversity (32% mean increase across sampling times) also affected the functionality of the bacterial communities present in the vermicompost. The diverse bacterial community showed enriched functional pathways related to soil health and plant growth, including the synthesis of antibiotics, amino acids, and phytohormones, as well as the degradation of bisphenol. In conclusion, in terms of microbial load and diversity, vermicompost derived from seaweed is a more valuable organic fertiliser than seaweed itself.

## 1. Introduction

Seaweed is a major contributor to ecosystem productivity in coastal ecosystems, and large amounts of seaweed appear seasonally in coastal areas as drift matter [1]. Removal of beach-cast seaweed is recommended because of the potential environmental or economic impacts caused by the accumulation of large amounts of dead biomass [2]. One of the easiest ways of managing seaweed waste is in agriculture, and farmers in coastal areas have used drift seaweed as a soil amendment and/or fertiliser, after prior treatment, such as rain washing and air drying [2]. However, the direct application of seaweeds to soil may have a deleterious impact on soil macrofauna, thereby negatively affecting the processes they contribute to nutrient cycling [3]. For example, the growth of soil earthworm species *Aporrectodea longa*, *A. caliginosa*, and *Lumbricus rubellus* is significantly impeded when they are fed exclusively with seaweed species *Laminaria digitata* and *Fucus serratus* [3].

The application of seaweed to soil has been demonstrated to have beneficial effects on soil properties and plant growth [4]. The beneficial effects of seaweeds can be attributed to high macro- and micronutrient content [5] and useful vitamin content [6]. Seaweeds have been shown to have biostimulant and biocidal properties, although most of these beneficial effects have been observed with seaweed extracts [7,8]. Nevertheless, the production of seaweed extracts entails an industrial process, which, in turn, generates waste. An alternative approach to dealing with drift seaweed is to utilise environmentally friendly processes that reduce, rather than increase, waste production, such as vermicomposting. Previous studies have demonstrated the viability of utilising seaweeds as a source for vermicomposting, with the production of high-quality organic fertilisers in terms of nutrient contents [9].

Vermicomposting is a process whereby the combined action of earthworms and microorganisms accelerates the decomposition of organic matter, significantly altering the physical, chemical, and microbiological properties of the resulting vermicompost [10]. The vermicomposting process comprises two distinct stages. Initially, the raw materials are digested by the earthworms, which then excrete the resulting material in the form of casts. The second stage involves the maturation of these casts [10]. A significant consequence of vermicomposting is the reduction in the volume of the substrate resulting from the combined action of microorganisms and earthworms. This is a crucial aspect of waste management policies [10]. Vermicomposting is expected to increase the nutrient content and availability due to the mineralisation of most of the organic compounds in seaweeds [2]. Previous research has shown the feasibility of vermicomposting seaweeds [11] and that vermicomposting enhances the mineralisation of processed seaweeds and increases microbial populations [12,13,14,15]. In addition, several studies have demonstrated enhanced microbial diversity and functional pathways associated with plant growth and health in vermicompost derived from plant residues such as grape marc [16,17,18], Scotch broom [19], and silver wattle [20] relative to the parent materials. These microbiological characteristics, rather than the nutrient content and availability, probably cause the known positive effects of vermicompost on plant growth and yield [21].

Although the microbiome of seaweed has been extensively studied [22], the microbiome of seaweed vermicompost has never before been analysed. In this study, we aimed to characterise the dynamics of bacterial communities of seaweeds during vermicomposting and to describe the changes in taxonomic groups and functions. We set up a vermicomposting reactor with a mixture of three seaweed species and analysed samples after 0, 7, 14, 21, and 28 days by high-throughput sequencing of 16S rRNA.

## 2. Materials and Methods

### 2.1. Seaweeds, Vermicomposting Set-Up and Sampling Design

Samples of different seaweeds (*Ulva lactuca*, *Ascophyllum nodosum*, and *Fucus spiralis*) were collected at low tide at the mouth of the river Miñouba (Domaio, Galicia, north-west Spain) in early April. The seaweed species were selected on the basis of their significant role in seaweed drift within the specified region [23]. The river current had already washed the seaweed, but they were soaked in tap water for one day before being added (in equal proportions) to the vermireactor to remove any excess salinity that would be harmful to the earthworms. The biochemical composition of the seaweed species differed significantly. The carbohydrate content was found to be 31.7–59%, 12.8–34.53%, and 34.7–76.8% for *A. nodosum*, *F. spiralis*, and *U. lactuca*, respectively [6]. The dietary fibre contents were 42.6, 49.1, and 54% for *A. nodosum*, *F. spiralis*, and *U. lactuca*, respectively [6], whereas their protein contents were 5.9–8.5, 7.1, and 9.3–17.1% for *A. nodosum*, *F. spiralis*, and *U. lactuca*, respectively [6].

The seaweeds were processed in a medium-scale vermireactor, as previously described [19]. The vermireactor, which was located in the greenhouse facilities of the Animal Ecology Group (GEA) of the University of Vigo (Spain), consisted of a 12 cm layer of mature vermicompost, which served as bedding material for the earthworms (*Eisenia andrei*). The initial earthworm population density in the vermireactor was 631 ± 15 individuals m^−2^, with a mean biomass of 300 ± 8 g m^−2^. The vermicompost bedding was covered with plastic mesh (holes 5 cm diameter), and a layer of seaweed (12 cm thick, 120 kg fresh weight, 60 kg of seaweed species) was placed on top of the mesh. The moisture content of the seaweed was maintained at approximately 85% throughout the experiment by covering the vermicomposting unit with a shading cloth. After 28 days, 10.2 kg of vermicompost was obtained (91% decrease in mass), and the density of the earthworm population was 1087 ± 12 individuals m^−2^, with a mean biomass of 445 ± 5 g m^−2^.

Samples of the vermicomposting process were taken at 0, 7, 14, 21, and 28 days. This was achieved by dividing the surface of the vermicompost reactor into five sections and randomly selecting five subsamples from each section to create a composite sample. The samples (25 samples, 5 per sampling time) were stored in Falcon tubes at −80 °C until analysis.

### 2.2. Amplification, Sequencing and Analysis of 16S rRNA Genes

DNA was extracted from samples (0.25 g fresh weight) using the MO-BIO PowerSoil^®^ kit according to the manufacturer’s protocol. The process was conducted under a laminar flow hood to prevent contamination. The V4 hyper-variable region of the 16S gene was sequenced using primers from the Earth Microbiome Project in a 2x250 Illumina MiSeq run (Illumina, San Diego, CA, USA). We used DADA2 (version 1.32.0) to infer amplicon sequence variants (ASVs), which are more accurate and reproducible than OTUs [24,25]. We followed the online DADA2 tutorial (https://benjjneb.github.io/dada2/tutorial.html, accessed on 23 July 2024) but modified the trimming parameters (forward reads truncated at 220 nt and reverse reads truncated at 130 nt). We used the Silva database (version 138.2) with the RDP naive Bayesian classifier with a bootstrap confidence level of 80 [26,27] to infer the ASV taxonomy. ASVs that remained unclassified at the phylum level (0.4% of sequences) were not considered further. Sequence data were deposited in the GenBank SRA database under accession number PRJNA1069356.

### 2.3. Bioinformatic and Statistical Analysis

All of the data were plotted and analysed using the phyloseq [28], tidyverse [29], RColorBrewer [30], patchwork [31], and vegan [32] packages implemented in R version 4.4.0 [33].

Prevalence filtering was conducted prior to the differential abundance tests, and only ASVs that were present in at least 10% of the samples were retained. This prevalence filtering step removed 58% of the ASVs but only 5% of the sequences. Rarefaction curves showed that the sampling depth for all samples was optimal for both the full dataset (3389 ASVs and 630,264 sequences) and the filtered dataset (1339 ASVs and 594,624 sequences, Supplementary Figure S1A,B).

UpSet plots were used to study the taxonomic turnover of seaweed bacterial communities during vermicomposting as implemented in the ComplexUpset R package [34]. UpSet plots are easier to interpret than Venn diagrams when there are more than three overlapping treatments. We defined common bacterial ASVs as those ASVs present in combinations of two, three, four, or five sampling times, and we defined specific ASVs as those present exclusively at each sampling time. In order to make the upset plot more informative, we have added bar charts to show the relative abundance of the ASVs per phylum for each comparison.

The differential abundance of ASVs and bacterial phyla and genera during seaweed vermicomposting was determined using negative binomial models [35]. The differential abundance of ASVs, as well as bacterial phyla and genera, was determined using Wald tests and *p*-values adjusted for false discovery rates (*p*-adj < 0.05). As multiple pairwise Wald tests were performed for each temporal comparison (0–7, 7–14, 14–21, and 21–28 days), the ‘raw’ *p*-values were further adjusted using the FDR method to correct for these multiple pairwise comparisons.

Taxonomic alpha-diversity was calculated as the number of observed ASVs, and diversity and richness were estimated using inverse Simpson and Chao1 indices, respectively. Phylogenetic alpha-diversity was calculated as Faith’s phylogenetic diversity [36]. For this purpose, raw data were rarefied 1000 times, and the average value was calculated for each diversity index using a custom R script. The effect of time (0, 7, 14, 21, and 28 days) on both the taxonomic and phylogenetic alpha-diversity of seaweed bacterial communities during vermicomposting was analysed by repeated measures ANOVA with the anova_test function in the rstatix package [37]. We chose to use this model because the data were balanced and sampled repeatedly in the same vermireactor. Post-hoc paired *t*-tests and corrected *p*-values were applied with the FDR method.

Taxonomic beta-diversity at the ASV level was estimated as the difference in the composition of the bacterial community between samples taken at different times during vermicomposting. Differences in beta-diversity were analysed at taxonomic (Bray-Curtis, Jaccard) and phylogenetic levels (weighted and unweighted Unifrac, [38]) by using permutational multivariate analysis of variance (PERMANOVA) on the transformed ASV table (variance stabilised transformation). When the overall PERMANOVA test indicated a significant difference, pairwise PERMANOVA tests were used to check for differences between different times of vermicomposting, and the *p*-values were corrected using the FDR method. PCoA was used to visualize changes in beta-diversity.

The functional composition of the metagenomes was predicted using the Phylogenetic Investigation of Communities by Reconstruction of Unobserved States software package [39]. We followed the same protocol described by Gómez-Roel et al. [40] to estimate and process data from PICRUSt2. As before, we focused the analysis on the pathways of amino acid biosynthesis, furfural and bisphenol degradation, antibiotic synthesis, antibiotic resistance, plant hormone synthesis, and nitrogen metabolism using the KEGGREST package (version 1.44.0) [41]. Prior to analysis, the PICRUSt2 output was standardised by z-scores, and a heatmap was then created [42]. The contribution of alpha- and beta-diversity was calculated using the FuncDiv library [43]. The effect of vermicomposting on pathway abundance and contributional diversity was analysed using repeated measures ANOVA and linear mixed models (nlme package, version 3.1-166 [44]), respectively, as the data generated by FuncDiv are unbalanced (i.e., not all KOs involved in a pathway are present at all sampling times). Pairwise post-hoc comparisons of pathway abundance and contributional diversity were performed using paired *t*-test and least squares means (emmeans package, version 1.10.6 [45]), respectively, and *p*-values were corrected using the FDR method.

## 3. Results

### 3.1. Changes in the Composition of Bacterial Communities

**Figure 1:**
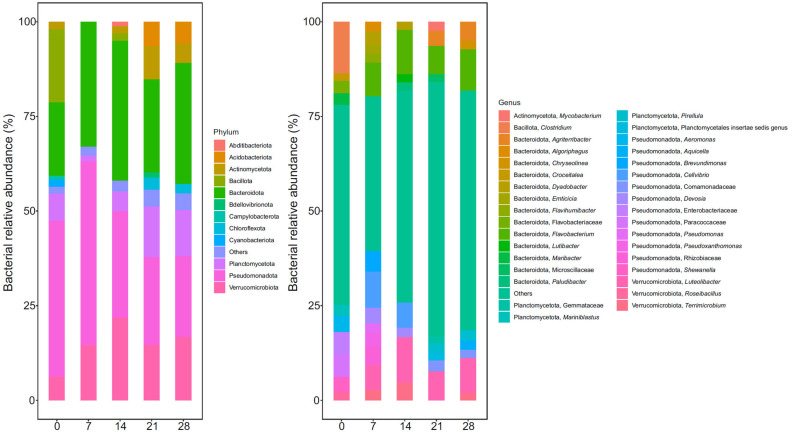
Relative abundance of bacterial communities at phylum and genus level during seaweed vermicomposting. The least abundant bacterial taxa (relative abundance < 1 and 2% for bacterial phyla and genera, respectively) were collapsed into ‘others’.

The seaweed-associated bacterial communities varied significantly during vermicomposting, and the changes were evident at the phylum, genus, and ASV levels. The major bacterial phyla detected were Pseudomonadota, followed by Bacteroidota and Bacillota (Figure 1). These three bacterial phyla showed different dynamics during vermicomposting. Thus, Pseudomonadota increased after 7 days and then decreased continuously (Figure 1, Supplementary Table S1). On the other hand, Bacteroidota increased after 7 days and then decreased with time, in contrast to the decrease-increase-decrease dynamic shown by Bacillota (Figure 1, Supplementary Table S1). The composition of the bacterial communities of seaweeds changed significantly at the phylum level early on in the vermicomposting process. The most notable changes were the elimination of several phyla (Bacillota, Actinobacteriota, and Campylobacterota), which readily occurred after seven days of vermicomposting (Figure 1, Supplementary Table S1). In subsequent days of vermicomposting, the main changes were related to increases in other phyla like Acidobacteriota, Bacteroidota, Bdellovibrionota, Chloroflexota, Planctomycetota, and Verrucomicrobiota (Figure 1, Supplementary Table S1). The changes were even more evident at the genus level. In this way, after seven days, there was a marked decrease in several bacterial genera being the higher decrease (log2-fold change lower than −8), those of *Terrisporobacter*, *Proteocatella*, *Granulosicoccus*, *Mariniblastus*, and *Clostridium* (Figure 1, Supplementary Table S2). Other bacterial genera like *Brevundimonas*, *Cellvibrio*, *Chryseolinea*, *Pseudoxanthomonas*, *Luteolibacter*, and *Terrimicrobium* showed marked increases after seven days of processing with log2-fold change values above 10 (Figure 1, Supplementary Table S2). After 14 days, the most prevalent trend was an increase in the abundance of bacterial genera, including *Formivibrio*, *Ilumatobacter*, *Pseudorhodoplanes*, and *Steroidobacter* (see Supplementary Table S2). After 21 days, a notable decline (log2-fold change values below −15) was observed in the abundance of certain bacterial genera, including *Bacteroides*, *Brevundimonas*, *Paludibacter*, and *Fusibacter* (Supplementary Table S2). Only six bacterial genera exhibited an increase in abundance after 28 days. At the ASV level, significant changes in 209, 145, 253, and 12 ASVs were observed between 0–7, 7–14, 14–21, and 21–28 days, respectively (Supplementary Table S3). Among these ASVs, the largest decreases occurred in ASVs from the genera *Clostridium*, *Cellvibrio*, *Bacteroides*, *Paludibacter*, and *Shewanella*, whereas the largest increases occurred in ASVs from the genera *Terrimonas*, *Pseudoxanthomonas*, *Luteolibacter*, *Prosthecobacter*, and *Chryseolinea* (Supplementary Table S3).

**Figure 2:**
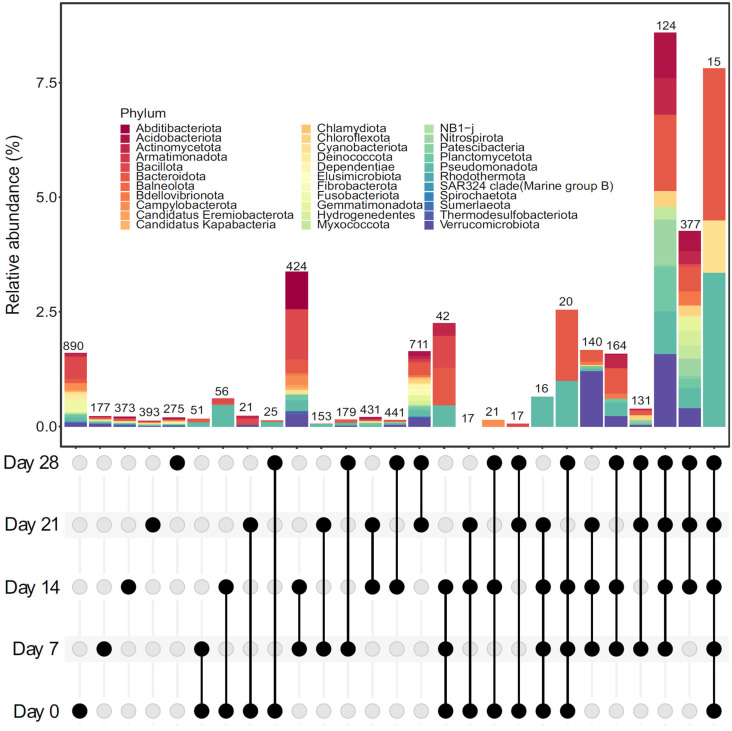
UpSet plot showing the unique and shared bacterial ASVs during seaweed vermicomposting. The mean relative abundances of unique and shared ASVs at the phylum level are shown. Black dots represent sampling times and lines indicate the intersections between sampling times.

Changes in bacterial composition were also evident when considering the number of unique bacterial ASVs (those occurring exclusively at each sampling time) and common bacterial ASVs (occurring at different sampling times) (Figure 2). For example, raw seaweed contained the largest number of unique bacterial ASVs, and the number decreased by up to 5 times when the earthworms began to process the seaweed (Figure 2). The changes in composition occurred rapidly, as very few (21 to 56) bacterial ASVs were common to the raw seaweed and vermicompost samples even after seven days (Figure 2). On the other hand, the earthworms promoted an increase in the similarity of bacterial communities over time, as shown by the high number of ASVs common to the vermicompost samples (Figure 2). Furthermore, the number of shared ASVs was higher between contiguous sampling times (7–14, 14–21, and 21–28) than between discontinuous sampling times (7–21, 14–28, etc., Figure 2), indicating a time-related increase in the similarity of the bacterial community composition.

### 3.2. Changes in the Diversity of Bacterial Communities

**Figure 3:**
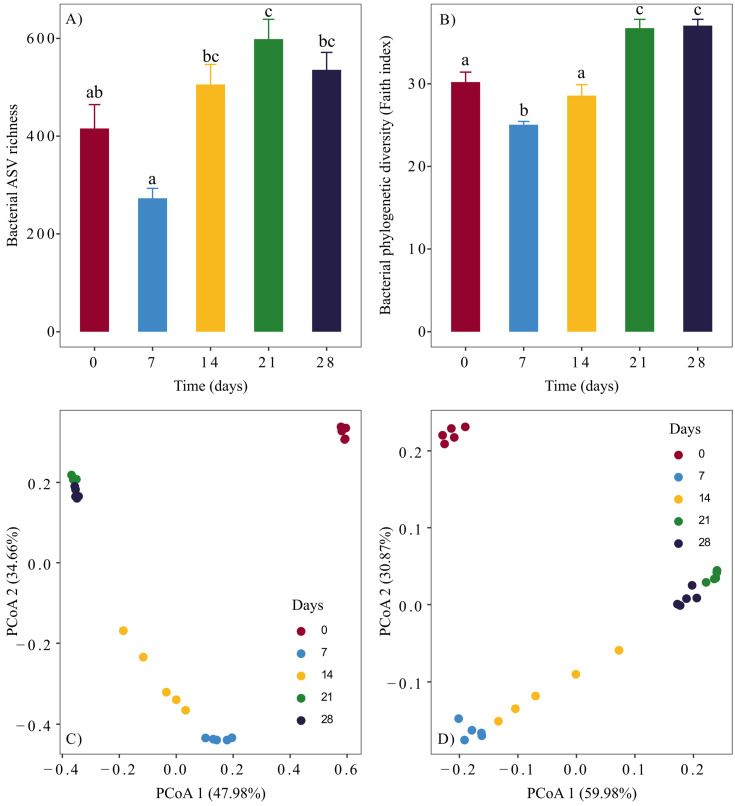
Changes in bacterial alpha diversity during seaweed vermicomposting, represented as (**A**) taxonomic amplicon sequence variant (ASV) and (**B**) phylogenetic diversity (Faith index). Different letters indicate significant differences between time points (paired *t*-test, FDR corrected). Changes in bacterial taxonomic and phylogenetic beta diversity during seaweed vermicomposting were determined by principal coordinate analysis of Bray-Curtis (**C**) and weighted UniFrac (**D**) distances, respectively.

The changes in the composition of bacterial communities resulted in significant changes in bacterial alpha diversity during seaweed vermicomposting. Bacterial alpha diversity increased significantly at both taxonomic (observed richness, Chao estimator, inverse Simpson index, Figure 3A, Supplementary Figure S2A,B, RM-ANOVA *p* < 0.0001 for all) and phylogenetic (Faith index, Figure 3B, RM-ANOVA *p* < 0.0001) levels. The increase occurred after 14 (Chao estimator and inverse Simpson index, Supplementary Figure S2) and 21 days (ASV richness and Faith index, Figure 2), after which the bacterial alpha diversity remained stable.

Similarly, vermicomposting altered the beta diversity of seaweed-associated bacterial communities significantly. Principal coordinate analysis showed that vermicomposting rapidly and strongly modified the beta diversity of seaweed-associated bacterial communities at both taxonomic and phylogenetic levels (PERMANOVAs *p* = 0.001 for all distance matrices, Figure 3C,D, Supplementary Figure S2C,D). Furthermore, the R^2^ values for the PERMANOVA test were very high in all cases (Bray-Curtis R^2^ = 0.87, unweighted unifrac R^2^ = 0.91, Jaccard R^2^ = 0.58, unweighted unifrac R^2^ = 0.79). In addition, the pairwise PERMANOVA tests revealed significant differences (*p* < 0.05) in all cases, except for the 21–28 days comparison for unweighted unifrac distances.

### 3.3. Changes in the Predicted Functional Composition of Bacterial Communities

**Figure 4:**
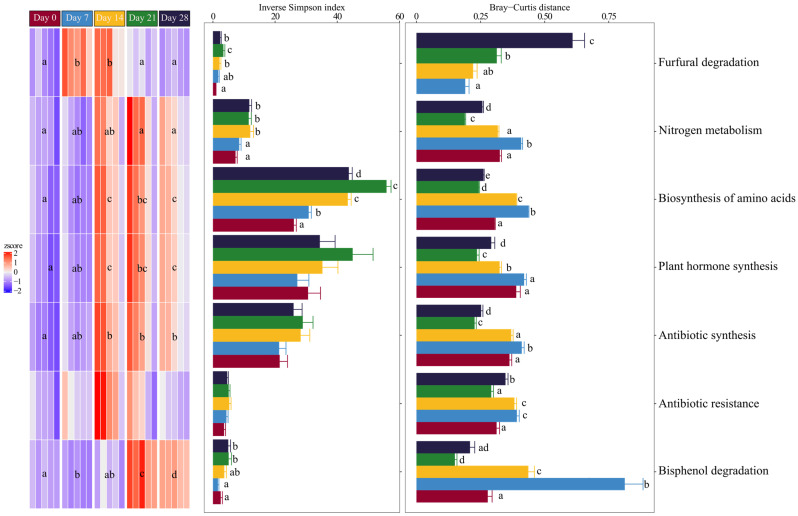
Heatmap of predicted bacterial community pathways and corresponding changes in contribution (i.e., bacterial ASVs with these functions), alpha diversity (inverse Simpson index), and beta diversity (Bray-Curtis distance) during seaweed vermicomposting. KEGG orthologue (KO) abundance data were z-score transformed. Different letters indicate significant differences between time points (paired *t*-test, FDR corrected).

Vermicomposting significantly modified the abundance of genes involved in all functional pathways analysed: antibiotic synthesis, amino acid biosynthesis, bisphenol and furfural degradation, nitrogen metabolism, and phytohormone synthesis (Figure 4, RM-ANOVA *p* < 0.05 for all pathways). Thus, the gene abundance of all pathways except furfural degradation and antibiotic resistance increased continuously and significantly over time (Figure 4). The contributional bacterial alpha-diversity increased significantly over time during the nitrogen metabolism, bisphenol and furfural degradation, and the amino acid biosynthesis pathways (mixed model *p* < 0.001 for all pathways, Figure 4). The contributional bacterial beta diversity varied significantly over time in all pathways analysed (mixed model *p* < 0.0001 for all pathways, Figure 4). However, each pathway yielded different temporal patterns. For example, furfural degradation increased consistently over time, whereas in the other pathways, the contributional bacterial beta diversity increased until day 7, then decreased, and finally increased after 28 days (Figure 4).

## 4. Discussion

### 4.1. Changes in the Composition of Bacterial Communities

Our data clearly indicates that seaweeds can be used as a source for vermicomposting. The vermicomposting process was completed in a relatively short period of time (28 days), yet despite this, there was a notable increase in earthworm population (73%) and biomass (50%), which resulted in a significant reduction in seaweed biomass (90%). The results demonstrate that earthworms are capable of processing and growing on seaweeds exclusively, despite previous vermicomposting studies [12,13,14,15] that have employed the mixing of seaweeds with animal manures and the unfavorable outcomes observed when feeding soil earthworms only with seaweeds [3]. The study findings clearly demonstrated that earthworms exerted a significant influence on the composition and diversity of the bacterial communities associated with seaweeds, even after only seven days. However, the most pronounced changes, i.e., the greatest differences in composition relative to raw seaweed, were observed at later stages of the vermicomposting process. Seaweed vermicomposting was faster than vermicomposting of distilled grape marc (1.5 times, [17]), silver wattle (2 times, [20]), scotch broom (3 times, [19]), Albariño (3 times, [18]), and Mencía grape marc (3 times, [16]). The differences in the speed of the process can be attributed to the effect of earthworm density. For example, seaweed vermicomposting was faster than vermicomposting of silver wattle (642 ± 46 earthworms m^−2^), Albariño grape marc (451 ± 33 earthworms m^−2^), and Mencía grape marc (270 ± 11 earthworms m^−2^). However, the seaweed vermicomposting process was faster than distilled grape marc and Scotch broom vermicomposting, despite a lower (1669 ± 136 earthworms m^−2^, distilled grape marc) or similar (834 ± 77 earthworms m^−2^, Scotch broom) earthworm density. This discrepancy may be due to differences in the chemical composition of the substrates, such as higher polyphenol or lignin content, which are more difficult to process [46].

The predominant bacterial phylum in raw seaweed samples was Pseudomonadota as in the other plant substrates like grape marc [16,17,18], silver wattle [20], Scotch broom [19], coconut leaves [47], and green waste [48]. However, notable differences were observed in the composition of other main bacterial phyla, which exhibited considerable variability across different plant substrates [16,17,18,19,20,47,48]. The variations were even more pronounced at the genus and ASV levels, resulting in a greater divergence in bacterial composition among the different types of vermicompost. However, in all cases, the composition of bacterial communities was rapidly altered during the initial stages of the vermicomposting process. The principal alteration in the bacterial composition of seaweeds resulting from earthworm activity was the elimination of several bacterial phyla (Bacillota, Actinobacteriota, and Campylobacterota) and an increase in the abundance of others (Verrucomicrobiota, Bacteroidota, and Acidobacteriota). This pattern has also been observed during the vermicomposting of other plant substrates, including grape marc [16,17,18], Scotch broom [19], silver wattle [20], coconut leaves [47], and green waste [48]. Remarkably, in these studies, the abundance of bacterial phyla decreased, but the bacteria were not eliminated. Compositional changes were also evident when the number of shared bacterial taxa at different sampling times was considered. Thus, shared bacterial taxa increased significantly over time, indicating an increase in compositional similarity over time, a pattern also previously described in the context of other plant substrates [16,17,18,19,20,47,48].

### 4.2. Changes in the Diversity of Bacterial Communities

The bacterial alpha diversity of the raw seaweed was lower (up to 3 times) than reported for fresh seaweed [49]. This is probably due to its origin in dead biomass and the washing protocol carried out before the start of vermicomposting. In contrast to other studies characterising the dynamics of bacterial communities during vermicomposting of plant materials, the taxonomic and phylogenetic alpha diversity values were higher in the seaweed-associated bacterial communities than in the bacterial communities associated with the parent materials, with the exception of grape marc from Mencía grapes [16], and green waste [48]. Despite this, and similar to findings on vermicomposting with other plant materials, we observed a continuous increase in alpha diversity during seaweed vermicomposting. Moreover, the alpha diversity of the seaweed vermicompost was similar, range 500–800 ASVs, for most of the plant substrates [16,17,18,19,20]. The readily decomposable green waste [48] and recalcitrant coconut leaves [47] yielded the highest and lowest diversity values (>3000 and 100 OTUs, respectively). It is noteworthy that microbial diversity exhibited an increase despite the continuous decrease in microbial biomass that is commonly observed during the process of vermicomposting [50]. The use of seaweed vermicompost, as well as other plant materials, should promote plant health owing to the known positive relationship between microbial diversity and soil health [51].

The vermicomposting process markedly modified the beta diversity of seaweed-associated bacterial communities, and the similarity between bacterial communities increased throughout the process. This is consistent with the findings of other vermicomposting studies involving plant substrates. The similarity of the communities at the final sampling times was even more pronounced, with samples from days 21 and 28 showing greater similarity (as evidenced by their closer positioning in PCoA plots) than samples from the initial sampling times (e.g., days 7 and 14). This effect was observed during the vermicomposting of silver wattle [20], distilled grape marc [17], and green waste [48] but not during the vermicomposting of other plant substrates. This could be due to the higher levels of shared taxa found in the final stages of vermicomposting during seaweed, silver wattle [20], and distilled grape marc [17] vermicomposting, which did not occur in other vermireactors.

### 4.3. Changes in the Predicted Functional Composition of Bacterial Communities

Vermicomposting of the seaweeds modified the composition and diversity of the bacterial communities originally present and also their functionality, as inferred by PICRUSt2. The functional pathways associated with the degradation of harmful compounds, such as bisphenol, increased significantly, as did those involved in the synthesis of beneficial compounds, such as phytohormones, amino acids, and antibiotics. For some of these pathways, there was an increasing, time-related trend in the contributional alpha-diversity, consistent with the overall increase in alpha-diversity. However, there was a time-related decrease in contributional beta diversity due to an increase in similarity observed with vermicomposting processing time. These results are consistent with those observed during the vermicomposting of sewage sludge [40] and other plant substrates [16,17,18,19,20]. This suggests that the effect of earthworms on microbial communities functioning during vermicomposting is a common phenomenon across different materials. This effect may be responsible for the beneficial effects of vermicompost on plant growth and yield [21].

## 5. Conclusions

The results of this study demonstrate the potential of vermicomposting seaweeds as a sustainable waste management solution. The high reduction rate (90% degradation of the initial material) and earthworm population growth indicate the feasibility of this approach. The present study sought to elucidate the taxonomic and functional dynamics of bacterial communities during the process of seaweed vermicomposting. The earthworms exerted a rapid and profound influence on the taxonomic and functional composition of the bacterial communities associated with seaweed. At the taxonomic level, the principal alterations were attributable to the elimination of bacterial phyla Bacillota, Actinobacteriota, and Campylobacterota and their associated bacterial ASVs. Consequently, a maximum of 4% of ASVs were shared between the raw seaweed and vermicomposting samples. Furthermore, the bacterial communities in the seaweed vermicompost exhibited greater diversity and functionality (up to 32 and 150%, respectively), with enriched pathways that directly promote plant growth through the production of phytohormones or mineralisation of nitrogen or indirectly through the synthesis of antibiotics, which may reduce pathogen growth. In conclusion, seaweed vermicompost may be regarded as a potentially valuable organic amendment, given its enhanced biofertiliser and biostimulant properties. Finally, vermicomposting of seaweed could be expanded to other seaweed species whenever their biochemical composition is similar to that of the species used in this study. One potential application could be the processing of the large amounts of Sargassum that drift to the shores every year.

## Data Availability

Sequences were uploaded to the GenBank SRA database under accession PRJNA1069356.

## Supplementary Materials

The following supporting information can be downloaded at: https://www.mdpi.com/article/10.3390/microorganisms13010030/s1, Figure S1: Rarefaction curves of the full and filtered datasets showing the number of amplicon sequence variants (ASVs) found in each sample during seaweed vermicomposting; Figure S2: Changes in bacterial alpha-diversity during seaweed vermicomposting, expressed as (A) taxonomic richness (Chao estimator) and (B) taxonomic diversity (inverse Simpson index). Different letters indicate significant differences between time points (paired *t*-test, FDR corrected). Changes in bacterial taxonomic and phylogenetic beta-diversity during seaweed vermicomposting were revealed by principal coordinate analysis of Jaccard (C) and unweighted UniFrac (D) distances, respectively; Table S1: Differential abundance in bacterial phyla during vermicomposting of seaweed analysed using negative binomial models as implemented in the package DESeq2 [35]; Table S2: Differential abundance in bacterial genera during vermicomposting of seaweed analysed using negative binomial models as implemented in the package DESeq2 [35]; Table S3: Differential abundance in bacterial amplicon sequence variants (ASVs) during vermicomposting of seaweed analysed using negative binomial models as implemented in the package DESeq [35].

## References

[1] Paine E.R., Schmid M., Boyd P.W., Diaz-Pulido G., Hurd C.L. (2021). Rate and fate of dissolved organic carbon release by seaweeds: A missing link in the coastal ocean carbon cycle. J. Phycol..

[2] Illera-Vives M., Seoane Labandeira S., Fernández-Labrada M., López-Mosquera M.E., Torres M.D., Kraan S., Dominguez H. (2020). Chapter 19—Agricultural uses of seaweed. Sustainable Seaweed Technologies.

[3] Butt K.R., Méline C., Pérès G. (2020). Marine macroalgae as food for earthworms: Growth and selection experiments across ecotypes. Environ. Sci. Pollut. Res. Int..

[4] Pei B., Zhang Y., Liu T., Cao J., Ji H., Hu Z., Wu X., Wang F., Lu Y., Chen N. (2024). Effects of seaweed fertilizer application on crops’ yield and quality in field conditions in China-a meta-analysis. PLoS ONE.

[5] Villares R., Fernández-Lema E., López-Mosquera E. (2013). Seasonal variations in concentrations of macro- and micronutrients in three species of brown seaweed. Bot. Mar..

[6] Xie C., Lee Z.J., Ye S., Barrow C.J., Dunshea F.R., Suleria H.A.R. (2024). A review on seaweeds and seaweed-derived polysaccharides: Nutrition, chemistry, bioactivities, and applications. Food Rev. Int..

[7] Deolu-Ajayi A.O., van der Meer I.M., van der Werf A., Karlova R. (2022). The power of seaweeds as plant biostimulants to boost crop production under abiotic stress. Plant Cell Environ..

[8] Santinon C., Ochi D., Beppu M.M., Vieira M.G.A. (2022). Chemical modifications in the structure of seaweed polysaccharides as a viable antimicrobial application: A current overview and future perspectives. Algal Res..

[9] Dang B.-T., Ramaraj R., Huynh K.-P.-H., Le M.-V., Tomoaki I., Pham T.-T., Hoang Luan V., Thi Le Na P., Tran D.P.H. (2023). Current application of seaweed waste for composting and biochar: A review. Bioresour. Technol..

[10] Domínguez J., Edwards C.A. (2004). State of the art and new perspectives on vermicomposting research. Earthworm Ecology.

[11] Patón D., García-Gómez J.C., Loring J., Torres A. (2023). Composting the invasive toxic seaweed *Rugulopteryx okamurae* using five invertebrate species, and a mini-review on composting macroalgae. Waste Biomass Valor..

[12] Ananthavalli R., Ramadas V., John Paul J.A.J., Karunai Selvi B., Karmegam N. (2019). Seaweeds as bioresources for vermicompost production using the earthworm, *Perionyx excavatus* (Perrier). Bioresour. Technol..

[13] Ananthavalli R., Ramadas V., John Paul J.A.J., Karunai Selvi B., Karmegam N. (2019). Vermistabilization of seaweeds using an indigenous earthworm species, *Perionyx excavatus* (Perrier). Ecol. Eng..

[14] Biruntha M., Karmegam N., Archana J., Karunai Selvi B., John Paul J.A.J., Balamuralikrishnan B., Chang S.W., Ravindran B. (2020). Vermiconversion of biowastes with low-to-high C/N ratio into value added vermicompost. Bioresour. Technol..

[15] Yatoo A.M., Bhat S.A., Ali M.N., Baba Z.A., Zaheen Z. (2022). Production of nutrient-enriched vermicompost from aquatic macrophytes supplemented with kitchen waste: Assessment of nutrient changes, phytotoxicity, and earthworm biodynamics. Agronomy.

[16] Gómez Brandón M., Aira M., Kolbe A.R., de Andrade N., Pérez-Losada M., Domínguez J. (2019). Rapid bacterial community changes during vermicomposting of grape marc derived from red winemaking. Microorganisms.

[17] Gómez-Brandón M., Aira M., Santana N., Pérez-Losada M., Domínguez J. (2020). Temporal dynamics of bacterial communities in a pilot-scale vermireactor fed with distilled grape marc. Microorganisms.

[18] Kolbe A.R., Aira M., Gómez-Brandón M., Pérez-Losada M., Domínguez J. (2019). Bacterial succession and functional diversity during vermicomposting of the white grape marc *Vitis vinifera* v. Albariño. Sci. Rep..

[19] Domínguez J., Aira M., Kolbe A.R., Gómez-Brandón M., Pérez-Losada M. (2019). Changes in the composition and function of bacterial communities during vermicomposting may explain beneficial properties of vermicompost. Sci. Rep..

[20] Rosado D., Ramos-Tapia I., Crandall K.A., Pérez-Losada M., Domínguez J. (2022). Grapevine treatment with bagasse vermicompost changes the microbiome of Albariño must and wine and improves wine quality. OENO One.

[21] Blouin M., Barrere J., Meyer N., Lartigue S., Barot S., Mathieu J. (2019). Vermicompost significantly affects plant growth. a meta-analysis. Agron. Sustain. Dev..

[22] Saha M., Dittami S.M., Chan C.X., Raina J.-B., Stock W., Ghaderiardakani F., Valathuparambil Baby John A.M., Corr S., Schleyer G., Todd J. (2024). Progress and future directions for seaweed holobiont research. New Phytol..

[23] Cremades J., Bárbara I., Veiga A.J. (2004). Intertidal vegetation and its commercial potential on the shores of Galicia (NW Iberian Peninsula). Thalasas.

[24] Callahan B.J., McMurdie P.J., Rosen M.J., Han A.W., Johnson A.J.A., Holmes S.P. (2016). DADA2: High-resolution sample inference from illumina amplicon data. Nat. Methods.

[25] Callahan B.J., McMurdie P.J., Holmes S.P. (2017). Exact sequence variants should replace operational taxonomic units in marker-gene data analysis. ISME J..

[26] Quast C., Pruesse E., Yilmaz P., Gerken J., Schweer T., Yarza P., Peplies J., Glöckner F.O. (2013). The SILVA ribosomal RNA gene database project: Improved data processing and web-based tools. Nucleic Acids Res..

[27] Wang Q., Garrity G.M., Tiedje J.M., Cole J.R. (2007). Naïve bayesian classifier for rapid assignment of rrna sequences into the new bacterial taxonomy. Appl. Environ. Microbiol..

[28] McMurdie P.J., Holmes S. (2013). Phyloseq: An r package for reproducible interactive analysis and graphics of microbiome census data. PLoS ONE.

[29] Wickham H., Averick M., Bryan J., Chang W., McGowan L., François R., Grolemund G., Hayes A., Henry L., Hester J. (2019). Welcome to the tidyverse. JOSS.

[30] Neuwirth E. (2022). RColorBrewer: Colorbrewer Palettes.

[31] Pedersen T.L. (2024). Patchwork: The Composer of Plots.

[32] Oksanen J., Simpson G.L., Blanchet F.G., Kindt R., Legendre P., Minchin P.R., O’Hara R.B., Solymos P., Stevens M.H.H., Szoecs E. (2022). Vegan: Community Ecology Package.

[33] R Core Team (2023). R: A Language and Environment for Statistical Computing.

[34] Krassowski M., Arts M., Lagger C. (2022). Max Complex-Upset.

[35] Love M.I., Huber W., Anders S. (2014). Moderated estimation of fold change and dispersion for RNA-seq data with DESeq2. Genome Biol..

[36] Faith D.P. (1992). Conservation evaluation and phylogenetic diversity. Biol. Conserv..

[37] Kassambara A. (2023). Rstatix: Pipe-Friendly Framework for Basic Statistical Tests.

[38] Lozupone C., Knight R. (2005). UniFrac: A new phylogenetic method for comparing microbial communities. Appl. Environ. Microbiol..

[39] Douglas G.M., Maffei V.J., Zaneveld J.R., Yurgel S.N., Brown J.R., Taylor C.M., Huttenhower C., Langille M.G.I. (2020). PICRUSt2 for prediction of metagenome functions. Nat. Biotechnol..

[40] Gómez-Roel A., Aira M., Domínguez J. (2024). Vermicomposting enhances microbial detoxification of sewage sludge, enabling potential application of the treated product in agroecosystems. Appl. Sci..

[41] Tenenbaum D. (2024). KEGGREST: Client-Side REST Access to the Kyoto Encyclopedia of Genes and Genomes (KEGG).

[42] Gu Z., Eils R., Schlesner M. (2016). Complex heatmaps reveal patterns and correlations in multidimensional genomic data. Bioinformatics.

[43] Douglas G.M., Kim S., Langille M.G.I., Shapiro B.J. (2023). Efficient computation of contributional diversity metrics from microbiome data with FuncDiv. Bioinformatics.

[44] Pinheiro J.C., Bates D.M. (2000). Mixed-Effects Models in S and S-Plus.

[45] Lenth R.V., Banfai B., Bolker B., Buerkner P., Giné-Vázquez I., Herve M., Jung M., Love J., Miguez F., Piaskowski J. (2024). Emmeans: Estimated Marginal Means, Aka Least-Squares Means.

[46] Liebeke M., Strittmatter N., Fearn S., Morgan A.J., Kille P., Fuchser J., Wallis D., Palchykov V., Robertson J., Lahive E. (2015). Unique metabolites protect earthworms against plant polyphenols. Nat. Commun..

[47] Gopal M., Bhute S.S., Gupta A., Prabhu S.R., Thomas G.V., Whitman W.B., Jangid K. (2017). Changes in structure and function of bacterial communities during coconut leaf vermicomposting. Antonie Leeuwenhoek.

[48] Cai L., Gong X., Sun X., Li S., Yu X. (2018). Comparison of chemical and microbiological changes during the aerobic composting and vermicomposting of green waste. PLoS ONE.

[49] Chen J., Zang Y., Yang Z., Qu T., Sun T., Liang S., Zhu M., Wang Y., Tang X. (2022). Composition and functional diversity of epiphytic bacterial and fungal communities on marine macrophytes in an intertidal zone. Front. Microbiol..

[50] Gómez-Brandón M., Fornasier F., de Andrade N., Domínguez J. (2022). Influence of earthworms on the microbial properties and extracellular enzyme activities during vermicomposting of raw and distilled grape marc. J. Environ. Manag..

[51] Banerjee S., van der Heijden M.G.A. (2023). Soil microbiomes and one health. Nat. Rev. Microbiol..

