# Supplementary material for: Taxonomic and Functional Dynamics of Bacterial Communities During Drift Seaweed Vermicomposting
Source: Microorganisms. 2024 Dec 27;13(1):30. doi: 10.3390/microorganisms13010030 (PMC11767599; doi:10.3390/microorganisms13010030)
Supplement: Supplementary file 1 [file microorganisms-13-00030-s001.zip › Supplementary_Figures.pdf]

A)

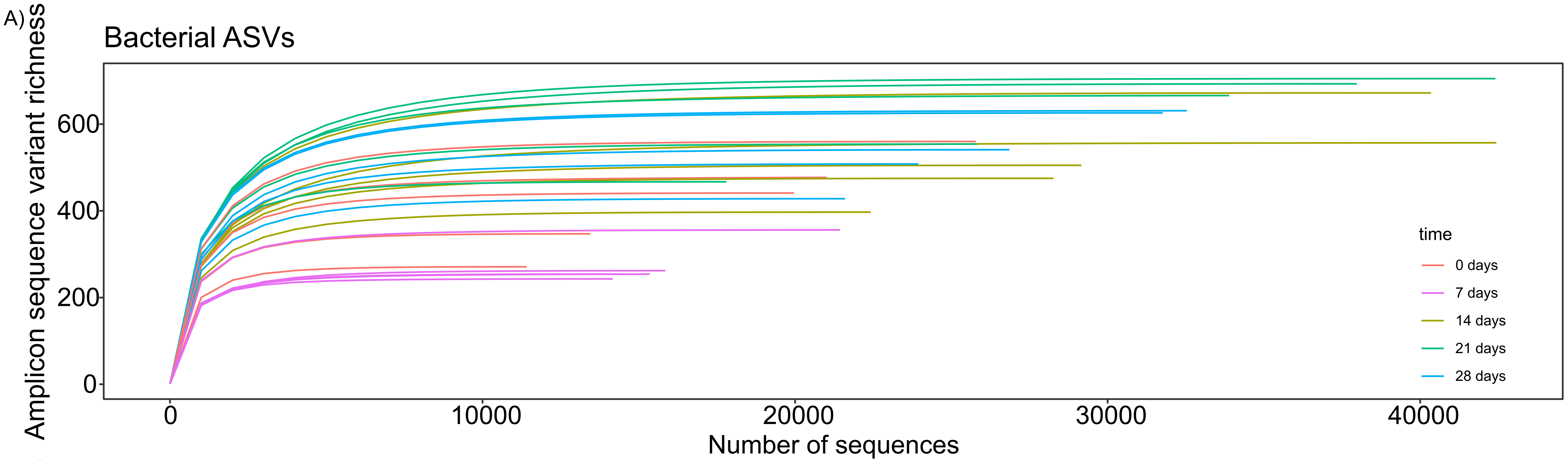

B)

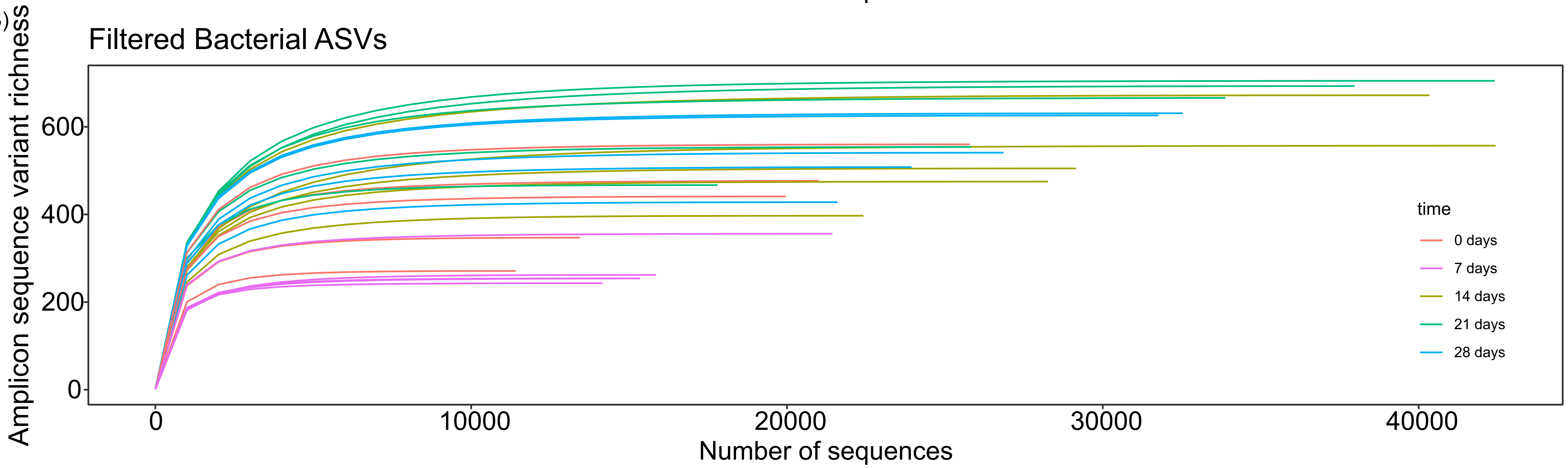

Figure S1: Rarefaction curves of the full and filtered datasets showing the number of amplicon sequence variants (ASVs) found in each sample during seaweed vermicomposting.

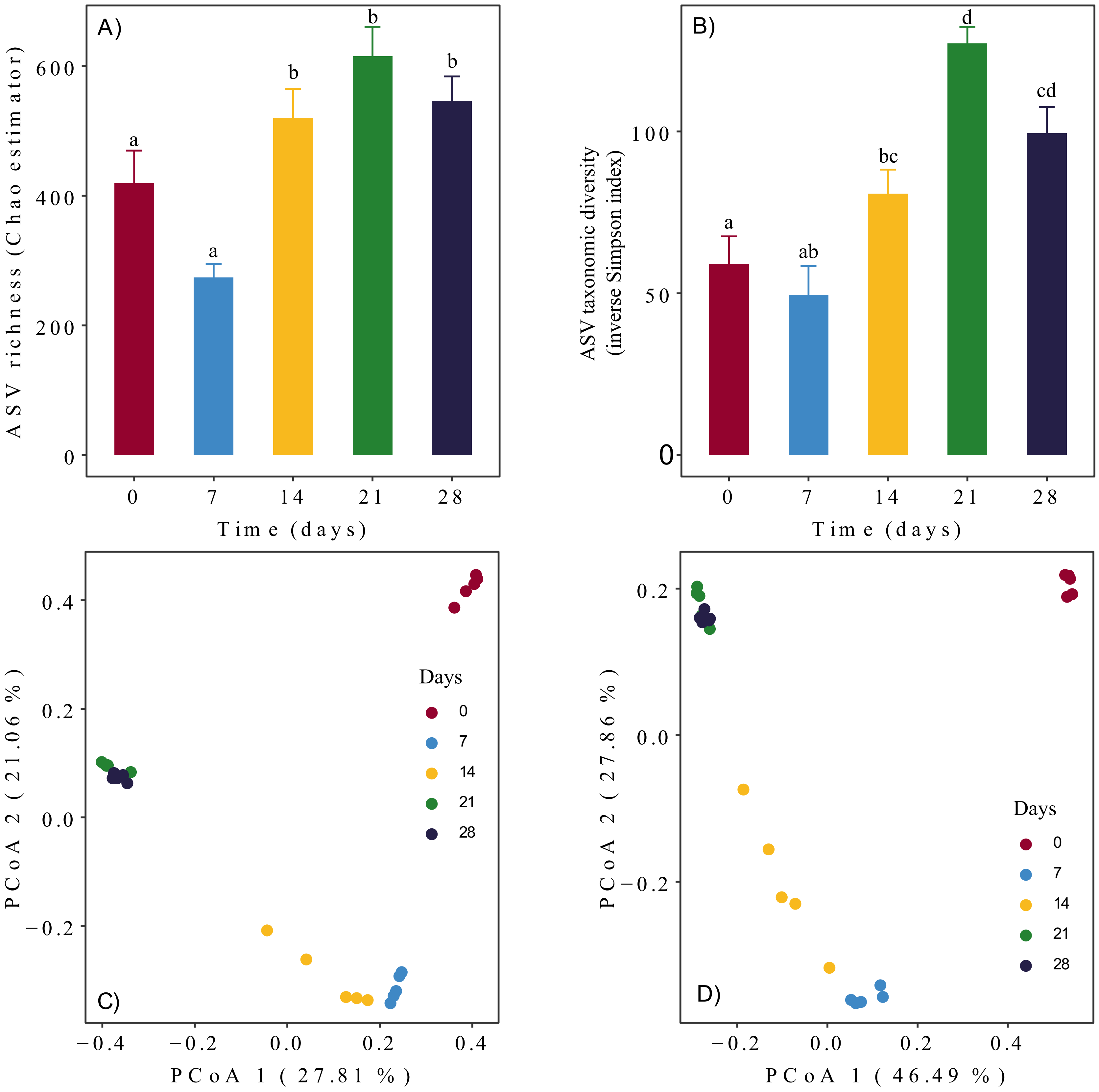

Figure S2: Changes in bacterial alpha-diversity during seaweed vermicomposting, expressed as (A) taxonomic richness (Chao estimator) and (B) taxonomic diversity (inverse Simpson index). Different letters indicate significant differences between time points (paired t-test, FDR corrected). Changes in bacterial taxonomic and phylogenetic beta-diversity during seaweed vermicomposting were revealed by principal coordinate analysis of Jaccard (C) and unweighted UniFrac (D) distances, respectively
